# Genetic characterization of TEM-type ESBL-associated antibacterial resistance in *Enterobacteriaceae* in a tertiary hospital in Ghana

**DOI:** 10.1186/s12941-016-0144-2

**Published:** 2016-05-04

**Authors:** Daniel Oduro-Mensah, Noah Obeng-Nkrumah, Evelyn Yayra Bonney, Ebenezer Oduro-Mensah, Kingsley Twum-Danso, Yaa Difie Osei, Sammy Tawiah Sackey

**Affiliations:** Department of Biochemistry, Cell and Molecular Biology, School of Biological Sciences, University of Ghana, Legon, Accra, Ghana; Department of Microbiology, University of Ghana Medical School, Korle-Bu, Accra, Ghana; Department of Virology, Noguchi Memorial Institute for Medical Research, University of Ghana, Legon, Accra, Ghana; Adabraka Polyclinic, Ghana Health Service, Accra, Ghana; Department of Applied Chemistry and Biochemistry, University for Development Studies, Navrongo Campus, Navrongo, Ghana

**Keywords:** Beta-lactamase, Inhibition zone chart, Conjugation, Reamplification

## Abstract

**Background:**

Antibiotic resistance due to the presence of extended-spectrum beta-lactamases (ESBLs) among *Enterobacteriaceae* is a worldwide problem. Data from Ghana regarding this resistance mechanism is limited. This study was designed to investigate the presence of TEM-type ESBL genes, their locations and their conjugabilities in clinical isolates of enterobacteria collected from the Korle-Bu Teaching Hospital in Ghana.

**Methods:**

Study isolates were characterized with respect to ESBL phenotype, TEM-type ESBL gene detection, location of the ESBL gene(s) and conjugability of the ESBL phenotype using nalidixic acid-resistant *Escherichia coli* K-12 as recipient. Phenotyping was by Kirby Bauer disk diffusion using cefpodoxime, ceftazidime, cefotaxime and their combinations with clavulanate. Gene detections were by PCR using *bla*TEM primers.

**Results:**

Overall, 37.96 % of 137 clinical isolates showed ESBL phenotype. The ESBLs occurred mostly in *Klebsiella* spp. (42.3 %) and then *Escherichia coli* (34.6 %). The TEM gene was detected in 48.1 % of ESBL-positive isolates and was determined to be plasmid-borne in 24 of 25 *bla*TEM detections. Overall, 62.7 % of TEM-producing isolates transferred the ESBL phenotype by conjugation.

**Conclusions:**

The results highlight the presence of TEM-type ESBLs in the Korle-Bu Teaching Hospital and show considerable risk of environmental contamination through the urine of infected persons. An inhibition zone chart was generated which indicates the possible presence of complex beta-lactamase types. The data points to the fact that the ESBL-producing bacteria may disseminate this resistance mechanism via conjugation.

## Background

The *Enterobacteriaceae* comprise a large family of clinically significant Gram-negative bacteria. They cause over 30 % of the morbidity and mortality associated with bacterial infections [1, 2] [CDC 2015, unpublished observation]. Resistance to *β*-lactam antimicrobials in *Enterobacteriaceae* has been due to largely the presence of *β*-lactamase enzymes [3, 4]. *β*-lactamase genes (*bla*) were originally found to be chromosomal [5]. Since the emergence of the first reports on plasmid-borne *blaTEM*-*1* in the 1960s, many more which appear to be mutants of the classic *TEM* genes, as well as novel types including SHV, CTX-M and OXA, have been found [6–8]. *β*-lactamase-mediated resistance may develop in vivo during chemotherapy, lending support to the prevalent view that ESBL plasmids are conjugative, may be borne on transposons, and that the genes may have high mutation frequencies [9, 10]. Also of concern is the increasing dissemination of ESBLs across hitherto wildtype species [11].

ESBL-producing bacterial isolates have been reported across Africa, even in isolated remote communities [11–16]. Beyond detection, data on specific identification of ESBLs are needed for deciding local therapy options, control strategies and recognition of unusual cases. In Ghana, investigations into mechanisms of *β*-lactam resistance have often been limited to phenotypic characterizations. This study reports the presence of TEM-type *β*-lactamase genes, their location(s) and their transferability by conjugation in clinical enterobacteria isolates collected at the Korle-Bu Teaching Hospital (KBTH).

## Methods

### Study isolates

Bacteria were isolated from clinical specimen (sputum, blood, feces, urine, cerebrospinal fluid, high vaginal swabs, wound swabs, pus) submitted to the Microbiology Laboratory of Korle-Bu Teaching Hospital (KBTH). A total of 137 isolates were included in the study. The isolates comprised only those recovered as members of the family *Enterobacteriaceae* [17] and identified as being responsible for patients’ clinical conditions. Conclusive identification of isolates was done with API 20E rapid test strips (bioMerieux SA, Marcy l’Etoile, France). Isolates were stocked in Luria–Bertani (LB)-ampicillin (60 μg/ml) broth containing 15 % glycerol and kept at −20 °C. All stocks were routinely plated to check for purity. Four National Collection of Typed Cultures (NCTC) isolates were included in the study as controls; NCTC 10418 (*β*-lactamase negative), NCTC 13352 (TEM 10 positive), NCTC 13353 (CTX-M-15 positive), NCTC 165032 (SHV-3).

### Detection of ESBL phenotype

Isolates were examined for ESBL phenotype by the Kirby-Bauer disk diffusion method of susceptibility testing and according to United Kingdom Health Protection Agency (HPA) guidelines (QSOP 51i2.2, 2008). Screening and confirmatory tests were done simultaneously on the same plate. Briefly, each test isolate was plated on Mueller–Hinton agar (Oxoid, UK). After incubating overnight at 37 °C, a 0.5 McFarland suspension in peptone broth (MAST, UK) of each was prepared. This suspension was then swabbed onto a cation-balanced Mueller–Hinton agar plate (MAST, UK) and left to dry completely. Antibiotic discs from a D52C ESBL detection kit (MAST, UK) were applied onto the plate and the setup was incubated at 37 °C for 18 h. Discs used were cefpodoxime (10 μg), ceftazidime (30 μg), cefotaxime (30 μg) and combination discs of cefotaxime/clavulanate (30/10 μg) and ceftazidime/clavulanate (30/10 μg). After incubation, inhibition zone diameters were measured and interpreted. A zone difference of ≥5 mm between the single and the combination disks for any of the antibiotics was regarded positive for ESBL production. *Klebsiella pneumoniae* ATCC 700603 was used as positive control for ESBL production.

### Assessment of antibiotics for ESBL phenotyping

The diagnostic performance of each antibiotic used for ESBL phenotyping was assessed on the following criteria: sensitivity, specificity, positive predictive value (PPV) and negative predictive value (NPV).

### Conjugation study

Only isolates confirmed as ESBL-producers were included in subsequent experiments. Conjugations were done using nalidixic acid^R^*E. coli* K-12 as the recipient strain. Three of the study isolates that had shown resistance to nalidixic acid were excluded from this stage of the study. Donor isolates were grown in LB-ampicillin (60 µg/ml) broth overnight. An aliquot of a pure stock of the recipient strain was also grown overnight in LB broth but with no antibiotic. For conjugation, aliquots of each donor and the recipient (1 ml each) were transferred into fresh LB broth and incubated for 2 h. Volumes of 500 µl of each donor culture were each taken and mixed with an equal volume of the recipient culture. The mixed cultures were incubated for 6 h at 37 °C. Selection for transconjugants was carried out on MacConkey agar (MAST, UK) supplemented with 32 µg/ml nalidixic acid and 100 µg/ml ampicillin [16]. Transconjugants were confirmed for ESBL production as previously described.

### Plasmid DNA extraction

Plasmid DNA was extracted from both ESBL-producing donor isolates and transconjugants according to previously described methods [18, 19]. For electrophoresis, 10 μl of each extract was mixed with 2 μl of 6X gel loading dye and electrophoresed on 0.7 % agarose gel stained with ethidium bromide (1 μg/ml). Electrophoresis was carried out in 1X Tris–acetate-EDTA (TAE) buffer at 10 V/cm for 1 h and visualized under UV transillumination [20]. The plasmid DNA samples obtained were used in subsequent PCRs.

### Total DNA extraction

Total DNA was also extracted from ESBL-producing donor isolates according to the method described by [21]. The DNA samples obtained by this procedure were used in subsequent PCR procedures.

### PCR for ESBL Genes

PCR amplifications of *bla*_TEM_, *bla*_SHV_ and *bla*_CTX-M_ genes were performed in 25 µl reaction mixes containing 25 units/ml of *Taq* DNA polymerase, 200 µM each of dATP, dGTP, dTTP and dCTP, 0.2 µM of each primer, 1.5 mM MgCl_2_ and 5 µl of plasmid or total DNA template. Amplifications were carried out with the following thermal cycling profile: initial denaturation for 10 min at 94 °C followed by 35 cycles of amplification consisting of 30 s at 94 °C, 1 min at the appropriate annealing temperature for the specific primer and 1 min at 72 °C for primer extension, and then 10 min at 72 °C for the final extension with a soaking step at 4 °C.

In cases where reamplifications of the previous PCR product were necessary, the reagent concentrations were modified to use only half the primer concentration used in the previous reaction and 1.0 µl of the previous product as template. Primers used and their corresponding annealing temperatures are shown in Table 1. For electrophoresis, 10 µl of each PCR product were prepared and run as already described.

**Table 1:** Sequences, annealing temperatures and expected product sizes of primer sequences targeting the specified ESBL genes

Gene	Primer	Annealing temp. (^°^C)	Expected product size (bp)
*bla*_TEM_	*f:* 5′-AAA CGC TGG TGA AAG TA-3′ *r:* 5′-AGC GAT CTG TCT AT-3′	45	720
*bla*_SHV_	*f:* 5′-ATG CGT TAT ATT CGC CTG TG-3′ *r:* 5′-TGC TTT GTT ATT CGG GCC AA-3′	60	726
*bla*_CTX-M_	*f:* 5′-GAC GAT GTC ACT GGC TGA GC-3′ *r:* 5′-AGC CGC CGA CGC TAA TAC A-3′	55	499

*Sources* Bonomo et al. [22] (*bla*_TEM_), Hanson et al. [23] (*bla*_CTX-M_), Bonomo et al. [24] (*bla*_SHV_)

## Results

A total of 137 clinical isolates belonging to *E. coli*, *Klebsiella* spp., *Citrobacter* spp., *Enterobacter* spp. and *Proteus* spp. were collected for this study. Table 2 shows the species distribution and clinical specimens from which isolates were recovered.

**Table 2:** Distribution of study isolates according to clinical sources

Isolate	Clinical source						Number of species	
	Urine	Blood	Sputum	CSF	HVS	Other^a^	Total	%
*Citrobacter spp.*	2	1	6	0	1	0	10	7.3
*Enterobacter spp.*	2	5	1	0	0	0	8	5.8
*Escherichia coli*	42	9	7	1	3	7	69	50.4
*Klebsiella spp.*	23	6	8	0	4	3	44	32.1
*Proteus spp.*	1	0	4	0	1	0	6	4.4
Total	70 (51.1 %)	21 (15.3 %)	26 (19.0 %)	1 (0.7 %)	9 (6.6 %)	10 (7.3 %)	137	100

Though only one instance of infection was observed from three CSF samples tested, it was separated due to the clinical significance of infection in the normally sterile cerebrospinal region

*CSF* cerebrospinal fluid, *HVS* high vaginal swab

^a^Refers to miscellaneous clinical sources (wound swabs, pus, aspirates, ear swabs)

### Detection of ESBL-producing isolates

**Fig. 1:**
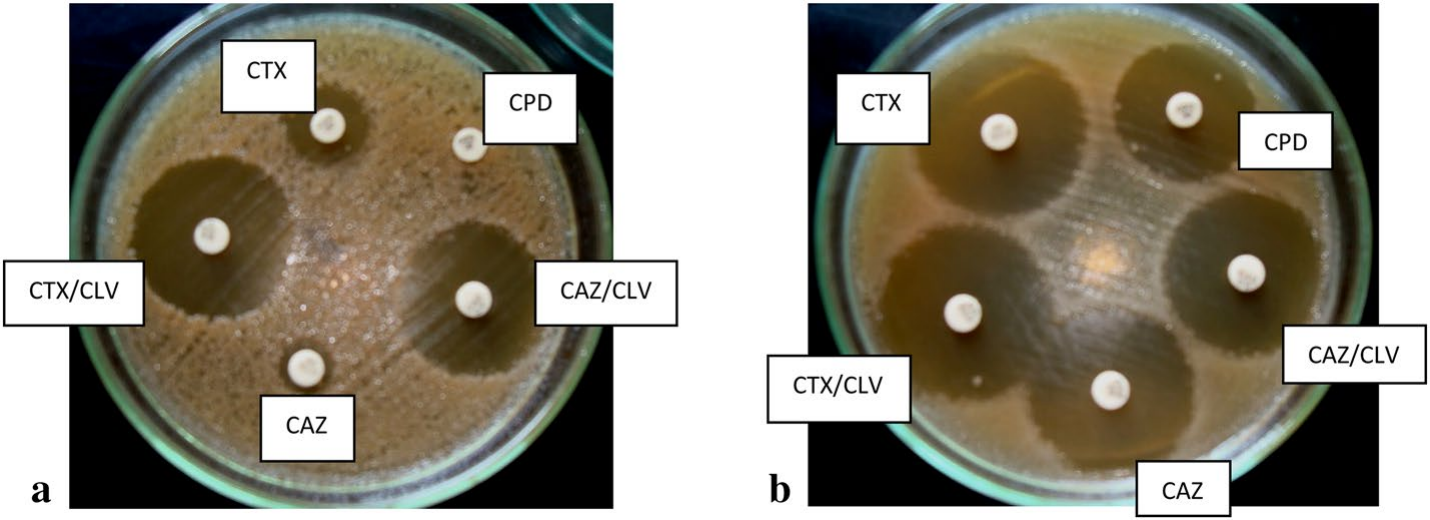
Appearance after over-night incubation for ESBL phenotyping of (**a**) an ESBL-positive strain and **b** an ESBL-negative strain. *Plate* **a** shows an example of what was taken as a positive ESBL test result. *Plate* **b** shows an example of a negative ESBL test result. In both cases, cefpodoxime was used only to screen and not in confirmation. Cefotaxime and ceftazidime were used in both screening and confirmation. *CPD* cefpodoxime 10 µg, *CPD/CLV* cefpodoxime 10 µg + clavulanate 10 µg, *CTX* cefotaxime 30 µg, *CTX/CLV* cefotaxime 30 µg + clavulanate 10 µg, *CAZ* ceftazidime 30 µg, *CAZ/CLV* ceftazidime 30 µg + clavulanate 10 µg

**Fig. 2:**
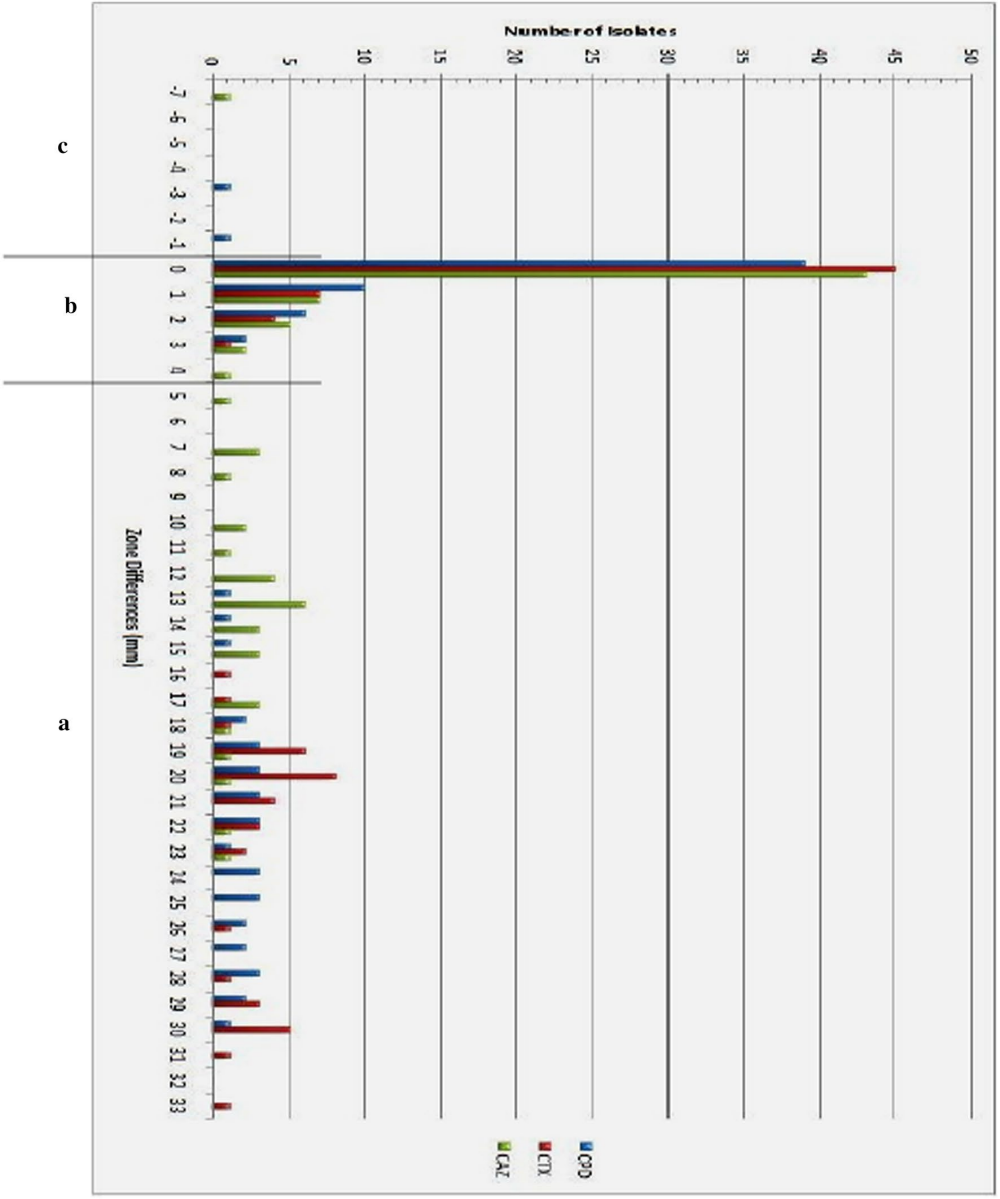
Inhibition zone chart for *Klebsiella* spp. and *E. coli* isolates. Zone differences observed between screening and confirmatory tests using each of the screening antibiotics alone and in its appropriate combination with clavulanate for confirmation. *CPD* cefpodoxime, *CTX* cefotaxime, *CAZ* ceftazidime. *Region* **a** zone differences for ESBL-positive phenotype. *Region* **b** zone-differences for ESBL-negative phenotype. *Region* (**c**) unusual zone differences

Figure 1 shows the setup for the disc diffusion assays for ESBL phenotype detection. Sensitivity values were 100 % for cefpodoxime (CPD), 100 % for cefotaxime (CTX) and 97.06 % for ceftazidime (CAZ). Cefpodoxime had highest specificity of 95.24 %, followed by CTX (94.64 %) and CAZ (92.98 %). Similarly, CPD recorded the highest PPD (92.98 %), then CTX (93.02 %) and CAZ (89.19 %). For NPV, CPD and CTX showed 100 %. Inhibition-zone size differences for *Klebsiella* spp. and *E. coli* isolates for all three antibiotics are presented in Fig. 2. Overall, 52 (37.96 %) of the 132 isolates included in this study expressed ESBLs (Table 3). The ESBL phenotype was most predominant in *Enterobacter* spp. (62.5 %) followed by *Citrobacter* spp. (60 %). Meanwhile, *Klebsiella* species comprised the majority of ESBL-producing isolates (42.3 %). Table 4 shows distribution of ESBL-producing isolates across clinical samples.

**Table 3:** ESBL prevalence amongst study isolates

Isolate	Not confirmed ESBL-positive^a^	ESBL prevalence	
		Confirmed ESBL-positive	% of overall prevalence
*Citrobacter* spp.	4	6 (60 %)	11.5
*Enterobacter* spp.	3	5 (62.5 %)	9.6
*Escherichia coli*	51	18 (26.1 %)	34.6
*Klebsiella* spp.	22	22 (50.0 %)	42.3
*Proteus* spp.	5	1 (16.7 %)	1.9
Total	85	52 (37.9 %)	100

ESBL prevalence was determined using MAST D52C combined discs by the Kirby-Bauer method of antibiotic susceptibility testing

^a^The column for “not confirmed ESBL-positive” includes isolates that failed screening tests as well as those isolates that may have passed the screening test(s) but failed confirmatory tests

**Table 4:** Distribution of ESBL-producing strains according to clinical sources

Clinical source	Sample size	ESBL phenotype present	
		Number	% of total number of ESBL-producers
Urine	70	31 (44.3 %)	59.6
Blood	21	9 (42.9 %)	17.3
CSF	1	0 (0)	0
HVS	9	3 (33.3 %)	5.8
Sputum	26	5 (19.2 %)	9.6
Other^a^	10	4 (40 %)	7.7
Total	137	52	100

*HVS* high vaginal swab, *CSF* cerebrospinal fluid

^a^Refers to miscellaneous clinical sources (wound swabs, pus, aspirates, and ear swabs)

### Conjugation studies

Results from the conjugation study indicated 64 % overall conjugability of ESBL genes amongst the ESBL-producing isolates (Table 5).

**Table 5:** Conjugabilities of ESBL-producer phenotype from isolates confirmed for ESBL production

Donor isolate	Total	Conjugation successful	Percent conjugability (%)
*Klebsiella pneumoniae*	22	19	86.4
*Escherichia coli*	18	9	50.0
*Enterobacter* spp.	5	2	40.0
*Citrobacter* spp.	4	1	25.0
*Proteus* spp.	1	1	100
Total	50	32	64

ESBL-positive study isolates were conjugated to nalidixic acid-resistant *E. coli* K-12. Selection of transconjugants was done on MacConkey agar (MAST, UK) supplemented with ampicillin and nalidixic acid

### ESBL genotyping

**Fig. 3:**
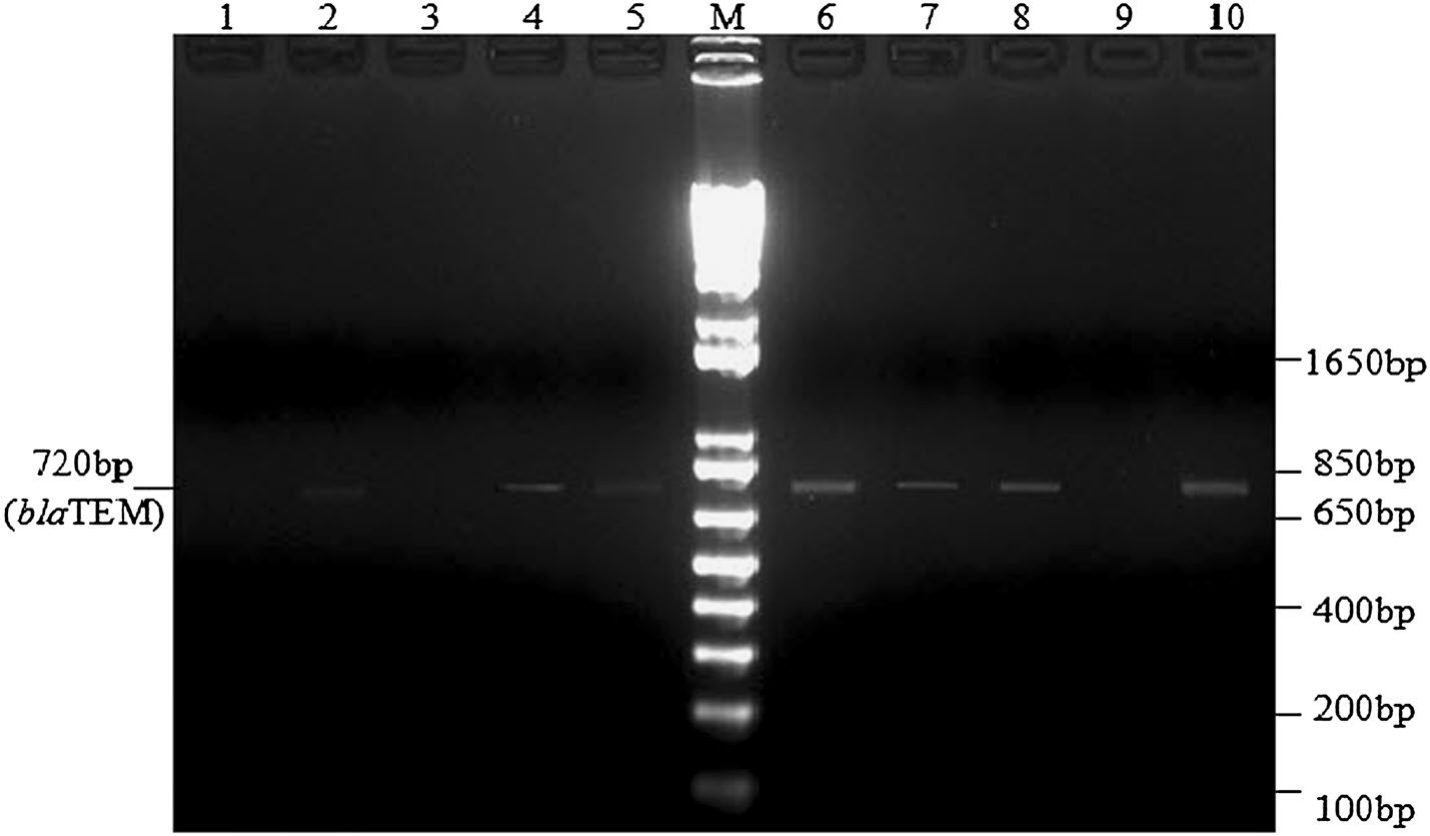
Bands observed after reamplification of previous PCR product with the TEM family-specific primers. PCR products were resolved on 2 % agarose gel stained with 1 µg/ml ethidium bromide at 100 V for 30 min. The gel was photographed under UV illumination. *Lane M* 1 kb plus DNA ladder, *Lanes 2, 4, 5, 7, 8, 10* PCR product from showing amplified *TEM* gene with band position at 720 bp, *Lane 6* TEM-3-producing *E. coli* NCTC 13351, *Lanes 1, 3, 9* PCR product with no amplification

The TEM gene was found in 25 (19.6 %) of the 127 isolates with ESBL-producing phenotype. None of the ESBL-positive isolates had SHV- or CTX-M-type genes (Fig. 3). Of the 25 strains with TEM-type genes, 12 (48 %) transferred their ESBL phenotype in conjugation assays (Table 6). In 24 (0.96 %) of the 25 TEM-gene positive strains, the gene was detected in both plasmid and total-DNA extracts.

**Table 6:** Numbers of detected conjugative TEM genes

Isolate	TEM	Number that had transferred by conjugation
*Citrobacter* spp.	0	0
*Enterobacter* spp.	1	0
*Escherichia coli*	11	3
*Klebsiella* spp.	12	8
*Proteus* spp.	1	1

The TEM primer produced PCR amplification product in DNA extracts from 25 of the ESBL-positive isolates. Verification of which of these genes had been transferred by conjugation was obtained by comparing this data to results of PCR on plasmid-DNA extracts from the transconjugants

## Discussion

Over the last decade, many studies have demonstrated the presence of ESBL-mediated resistance in bacteria causing infections in patients [25–28]. Despite several reports of ESBL presence in Ghana, the characterization of ESBL genotypes in relation to chromosomal or plasmid locations—and their transferability by conjugation—are not described.

### ESBLs in study isolates

The study revealed a moderate [37.96 % (n = 52/137)] prevalence of ESBL phenotype among the study isolates. In Table 3, the column for those that were not confirmed positive included all the bacteria that failed the screening tests as well as those that may have passed a screening test but failed all three confirmatory tests. The ESBL-producing isolates were found among every genus tested.

Two observations merit attention. First, the level of ESBLs is comparable to that reported by other ESBL-affected institutions in Ghana [29, 30] but higher than figures reported in many other reviews spanning across several regions [25–27]. In the African context however, the situation in KBTH appears to be rather moderate [13, 14, 30]. If current trends of lack of routine ESBL-monitoring and lack of ESBL-control strategies continue however, it can be expected that the prevalence will rapidly increase. This will lead to increased treatment expenses, longer hospital stays and possibly, mortality. Second, our results point to ESBL-producing isolates as being present among various members of the family *Enterobacteriaceae* and not just *Escherichia coli* and *Klebsiella* spp. In contrast to the attention paid mostly to ESBLs in *Escherichia coli* and *Klebsiella* spp. [25–28], the study showed that although a minority of the isolates were non-*Escherichia coli* and non-*Klebsiella* species, >40 % of these were ESBL-producers.

It is also noteworthy that ESBL detection was considerably high among *Citrobacter freundii* (>50 %) and *Enterobacter cloacae* (>60 %). Though only six *Proteus* spp. isolates were included in the study, their producer prevalence of 16.7 % cannot be over-looked. Therefore, the practice of not investigating ESBL presence in these pathogens may have an adverse impact on patients who are treated with extended-spectrum cephalosporins. With their hypermotility and ability to easily dominate biofilms, ESBL-producing *Proteus* spp. is a very uncomfortable prospect.

ESBL-producing strains appeared to be most common amongst isolates cultured from urine samples. Considering the fact that some bacteria will certainly be expelled in situ when urine is excreted, this has rather significant implications for risk of environmental contamination and cross-infections.

### Performance of detection agents

Extent of reliability of phenotypic screening and confirmatory agents is needed to confidently rule out isolates that do not pass screening and/or confirmatory tests. Both cefpodoxime and cefotaxime had 100 % sensitivity scores and 100 % negative predictive value (NPV) scores. After comparing specificity and positive predictive value (PPV) scores however, it was evident that cefotaxime had a lesser likelihood of refusing a possible-positive than cefpodxime. It is generally accepted that cefpodoxime is the best single agent for ESBL detection [31]. From these results however, cefotaxime, with its slightly lower specificity score but higher positive predictive value score, performed just as well as, if not better than, cefpodoxime. Data of this nature are very important since for a given study involving large numbers of samples, one cannot screen all collected isolates by genotypic methods which are the gold standard in ESBL detection.

Unusual inhibition zone patterns should however be treated on a case-by-case basis.

### Inferences from inhibition zones

Comparing the cefpodoxime zone size differences represented in Fig. 2 to data from the British society for antimicrobial chemotherapy [32], certain implications were realised. It was seen that the patterns of size differences represented in both figures were quite different. In the reference data, zone differences of the majority of ESBL-producer isolates fell between the ranges of 6–26 mm whereas from this study, zone differences fell between 13–30 mm with the majority ranging from 18 to 30 mm. The relatively higher zone sizes realized from this data may be indicative of differences in plasmid copy numbers and activity profiles of the enzymes as well as differences in permeability characteristics of the isolates from the two different localities.

Two *Klebsiella* isolates had cefpodoxime zone size differences that were in the negatives; −1 and −3 (Fig. 2). The lack of synergistic effect with the addition of clavulanate might suggest that they produce AmpC enzymes. However, they both tested screen-negative with each antibiotic used, uncharacteristic of AmpC-producing strains. Comparing to the reference data, those isolates were likely to produce K1 β-lactamases. The K1 enzymes are a family of β-lactamses, encoded by chromosomal genes, and which are presently thought to be unique to *Klebsiella oxytoca*. The two isolates however had corresponding ceftazidime zone-size differences of −7 and 1 mm. This implies that any K1 presence in them is unlikely since ceftazidime is notably susceptible to K1 enzymes. Also, the activity of K1 enzymes on ceftazidime is greatly diminished with the addition of clavulanate [33, 34]. Both isolates were amongst those most susceptible to the screening agents used, including ceftazidime, but were obviously not affected by clavulanate synergy. Currently, the only β-lactamase class that fits this profile of decreased affinity for β-lactam substrates and non-inhibition by clavulanate is the mutants of the inhibitor-resistant TEMs (IRTs) which belong to class A, subgroup 2br. The 2br subgroup contains the inhibitor-resistant TEM-type enzymes that evolved from the classical TEMs by acquiring the substitutions M69 V and N276D [29]. Derivatives of these enzymes are the complex TEM mutants (CTMs) that combine inhibitor-resistance with decreased affinity for cephalosporins [35].

A few isolates (4 for cefpodoxime, 3 for cefotaxime and 5 for ceftazidime) were screen-positive but failed the confirmatory tests, with 2 strains common to cefpodoxime and cefotaxime and 1 strain being common to all three antibiotics. This kind of phenotype, though typical of ampC-producers, may also be expressed by strains with CTMs, high levels of the TEM-1 enzyme and OXA-type ESBLs.

An interesting observation was one *Klebsiella* isolate which was clearly screen-positive with cefpodoxime, tested screen-negative for ceftazidime but was affected by clavulanate synergy with ceftazidime. Screening by ceftazidime alone would have led to the isolate being ruled as an ESBL non-producing strain. However, the observed synergy upon addition of clavulanate suggests, besides the possibility of AmpC-production, the presence of other β-lactamase types that are susceptible to the inhibitor.

It is seen from Fig. 2 that from 5 to 15 mm in region (a), inhibition of cefotaxime hydrolysis by clavulanate is not shown. This is a likely indication of the presence of CTX-M enzymes which preferentially hydrolyse cefotaxime relative to cefpodoxime and ceftazidime. Like all other ESBLs, CTX-M enzymes are susceptible to inhibition by clavulanate. The effect of clavulanate synergy with cefpodoxime and ceftazidime is seen but since CTX-M’s normally hydrolyse these with relatively lower turn-over rates, the zone size differences there are not as large as for cefotaxime which the enzymes would hydrolyse with greater efficiency in the absence of clavulanate.

### Conjugation study

In the present study, about 63 % conjugability was observed, higher than the 38.7 % reported in Cameroon by [36]. In contrast to the report by [37] where *E. coli* showed higher ESBL conjugability than *Klebsiella* spp., *Klebsiella* had the highest rate of 86.4 % followed by *E. coli’s* 50 % (Table 5). This pattern of results may stem from the fact that *Klebsiellae* have been found to frequently harbor plasmids bearing *bla*_SHV_ -type genes, and considering the usual sizes of *bla*_TEM_—(less than 80 kb) and *bla*_SHV_—(from about 80 kb to up to 350 kb in some *Klebsiella* isolates) bearing plasmids, the latter are probably more likely to be conjugable.

### DNA extraction and PCR

All isolates positive for ESBL-production were selected for DNA extraction. Attempts at linearization of plasmids using *Pst*I, *Hin*dIII and *Eco*RI restriction enzymes always failed. A plasmid which consistently appeared in the 22 kb position (data not shown) was found to be present in some of the plasmid extracts in which TEM genes were detected. Further resolution of the 22 kb band showed that it consisted of two clearly distinct bands placed closely together. All the plasmid extracts had the lower band. Plasmid preparations from a few of the transconjugants showed only the lower band, suggesting that the higher band may have been a non-conjugable plasmid species.

DNA extracted by a Promega Plus SV Miniprep DNA kit and [20] were used for PCR with *bla*TEM, *bla*SHV and *bla*CTX-M primers. Initial PCR rounds on control isolates showed amplification products for *bla*TEM but none for *bla*SHV and *bla*CTX-M positive controls (data not shown). Generally, the *bla*_TEM_ products required re-amplification to be clearly visible (Fig. 3). A total DNA extract belonging to an *Enterobacter* spp. isolate produced amplification product but nothing was observed with its corresponding plasmid extract. This suggested that the detected gene may have been chromosomal. With the *Klebsiella* spp. and *E. coli* isolates that produced amplicons with both DNA extracts, there is at least a plasmid-borne *bla*_TEM_ gene present even if there may be others that are chromosomal. Particularly with the *Proteus* spp. DNA sample, the detection made with the total-DNA extract was notably very faint relative to that from its plasmid extract. Due to the fact that any plasmid template present would be very dilute in the total-DNA preparation, this strongly suggested again that the gene detected by PCR may have been plasmid-borne in the *Proteus* isolate.

### Plasmid mediation and conjugability

TEM-producing isolates that did not have the 22 kb plasmid included 2 *E. coli*, 1 *Enterobacter* and 1 *Proteus* isolate. This may have been a TEM-bearing plasmid since it was common to 84 % of the isolates in which TEM was detected. Judging from its size however, it was unlikely to be conjugative and this had been demonstrated by its absence in the plasmid extracts from the transconjugants. It was unlikely also to be mobilisable since it was not observed in any of the transconjugants tested. [38] has however previously reported the successful conjugation of a 10 kb ESBL-associated resistance determinant. The *Proteus* isolate had transferred its phenotype during the conjugation study (Table 6), suggesting that the gene it carried was on a mobilisable plasmid. The proportion of ESBL conjugability reported here also suggests that precautions should be taken to minimise the possibility of horizontal transfer of ESBL genes as well as clonal spread as reported by [39].

## Conclusions

Detection level of ESBL-producing strains amongst the isolates studied was 37.96 % (n = 137). It is important that subsequent work carried out on ESBL-producing strains should take ampC *β*-lactamase detection as well as influence from other types of β-lactamases that may be present into account. This is because as more and more isolates begin to concomitantly produce these enzymes, interactions between them will make antibiograms of the isolates rather difficult to interpret. In the majority of cases, ESBL-producing bacteria in the hospital could transfer the phenotype by conjugation. Interpretative reading of the inhibition-zone chart suggests that other types of β-lactamases such as inhibitor-resistant TEMs (IRTs) and ampCs might have been present amongst the isolates. These need to be investigated.

## Abbreviations

AmpC: ampicillin-hydrolyzing cephalosporinase
bla: beta-lactamase gene
ESBL: extended spectrum β-lactamase
M: methionine
V: valine
N: asparagine
D: aspartate
